# Structured navigation in a goal-directed task reveals flexible spatial coding

**DOI:** 10.64898/2026.07.27.741044

**Published:** 2026-07-28

**Authors:** Tucker G Fisher, Marielena Sosa, Alexander Gonzalez, Xiaotong Cheng, Lisa M Giocomo

## Abstract

Animal survival depends on accurate navigation. The last several decades have revealed that neurons encoding position and orientation can support navigation through the construction of an internal map of space for the external world. However, current experimental approaches for studying this system require tradeoffs between behavioral richness and experimental control; open field tasks often lack structured trials or trajectories and virtual reality paradigms constrain natural movement variables. Here, we introduce a platform that combines freely moving behavior with control over environmental stimuli, trajectories, and reward locations. This platform is compatible with chronic Neuropixels recordings, dynamic optical projection of visual stimuli and real-time pose estimation. Using this platform, we show rats can learn a multistage spatial targeting task and that high density Neuropixels recordings can be performed in conjunction. Recordings in medial entorhinal cortex revealed canonical spatial and head direction tuning, along with population-level activity that tracked distance to task-relevant goals. Together, this platform provides a powerful approach for examining the neural basis of flexible navigation under experimentally controlled yet naturalistic conditions.

## Full Text Availability

The license terms selected by the author(s) for this preprint version do not permit archiving in PMC. The full text is available from the preprint server.

